# *Brassica nigra* L. Seed Extracts as a Promising Source of Antioxidant and Anti-Inflammatory Agents

**DOI:** 10.3390/ijms27104603

**Published:** 2026-05-20

**Authors:** Nichcha Nitthikan, Siripat Chaichit, Kanittapon Supadej, Jintana Junlatat, Kanokwan Kiattisin

**Affiliations:** 1 Office of Research Administration, Chiang Mai University, Chiang Mai 50200, Thailand; nichcha.n@cmu.ac.th; 2 Department of Pharmaceutical Sciences, Faculty of Pharmacy, Chiang Mai University, Chiang Mai 50200, Thailand; siripat.chaichit@cmu.ac.th; 3 Department of Medical Technology, School of Allied Health Sciences, University of Phayao, Phayao 56000, Thailand; kanittapon.su@up.ac.th; 4 Faculty of Thai Traditional and Alternative Medicine, Ubon Ratchathani Rajabhat University, Ubon Ratchathani 34000, Thailand; jintana.j@ubru.ac.th

**Keywords:** back mustard seed, *Brassica nigra*, antioxidant, anti-inflammation, antibacterial, interleukin-6, interleukin-31, sinapic acid, molecular docking

## Abstract

Black mustard (*Brassica nigra* L.) seeds are a rich source of bioactive phytochemicals; however, their antioxidant, antibacterial, and anti-inflammatory potential has not been comprehensively explored. Therefore, this study aimed to assess antioxidant, antibacterial, and anti-inflammatory effects of black mustard seed extracts obtained from Soxhlet extraction with hexane (HE) and ethanol (EE), and ultrasonic-assisted ethanolic (UE) extraction. HPLC analysis confirmed the presence of sinapic acid in all extracts. Phytochemical profiling revealed that the EE was enriched in phenolic compounds, while the UE exhibited a higher flavonoid content. Accordingly, both EE and UE demonstrated strong antioxidant activities, including radical scavenging capacity, reducing power, and inhibition of lipid peroxidation. All extracts demonstrated antibacterial activity against *Staphylococcus epidermidis*. The anti-inflammatory potential of extracts was supported by the inhibition of lipoxygenase and protease. The UE showed the strongest lipoxygenase inhibition, while the EE and UE exhibited comparable protease inhibitory effect. Regarding RAW264.7 cells, the extracts were non-cytotoxic and reduced the expression of IL-6 and IL-31. Molecular docking analysis suggested that sinapic acid contributes to the anti-inflammatory activity through interactions with key inflammatory targets. Overall, the EE and UE demonstrated multitarget antioxidant, antibacterial, and anti-inflammatory activities, supporting their potential application in functional and dermatological formulations for inflammation management.

## 1. Introduction

The use of medicinal plants for the treatment of various skin conditions has been widely recognized for decades. Natural products derived from plant extracts have been extensively used for the management of several diseases, particularly inflammatory disorders [1]. Mustard seeds, belonging to the Brassicaceae family, are valued not only as culinary spices but also as rich sources of bioactive phytochemicals with pharmacological potential. Traditionally, mustard seeds have been used as digestive stimulants, laxatives, antiseptic agents, and topical remedies for skin disorders, rheumatic pain, and neuralgic conditions in many regions, including South Asia, East Asia, and the Middle East [1,2,3]. Among mustard species, black mustard (*Brassica nigra* L.) is widely cultivated and is particularly rich in sinigrin, a glucosinolate precursor of allyl isothiocyanate (AITC), as well as phenolic acids such as sinapic acid and ferulic acid [4]. Black mustard is also commercially available in many regions worldwide, supporting its potential for large-scale utilization as a source of bioactive compounds. Sinapic acid, one of the major phenolic constituents, has been widely reported for its strong antioxidant and anti-inflammatory properties, mainly through free radical scavenging and suppression of inflammatory signaling pathways including nuclear factor kappa B (NF-κB), which plays a key role in cytokine production [5]. In recent years, sinapic acid has attracted increasing attention due to its diverse biological activities, including antimicrobial, antioxidant, antilipidemic, and anti-inflammatory properties [6,7].

Inflammation is a complex and dynamic immune response that is essential for maintaining tissue homeostasis and protecting the host against infection, injury, and external stimuli. While acute inflammation is necessary for eliminating pathogens, dysregulated or persistent inflammation can contribute to chronic disorders affecting both skin and immune system [8]. At the molecular level, cytokines play central roles in coordinating innate and adaptive immune responses, with macrophages serving as major sources of inflammatory mediators such as tumor necrosis factor alpha (TNF-α), interleukin 6 (IL-6), IL-31, nitric oxide (NO), and cyclooxygenase-2 (COX-2) [9]. In inflammatory skin conditions, microbial imbalance on the skin surface can further amplify cytokine-mediated immune responses. Skin-associated bacteria such as *Staphylococcus epidermidis* may influence cutaneous inflammation, although typically considered a commensal microorganism, may contribute to cutaneous inflammation when overgrown. Excessive colonization of *S. epidermidis* can disrupt microbial homeostasis and stimulate skin cells to release inflammatory mediators, thereby aggravating redness, irritation, and pruritic responses [10]. Among these mediators, IL-31 has gained increasing attention due to its strong association with pruritus, atopic dermatitis, and epidermal barrier dysfunction. Previous studies have shown that extracts derived from mustard species possess notable antioxidant and anti-inflammatory activities, largely attributed to glucosinolate metabolites and phenolic constituents [11]. These bioactive compounds have been reported to suppress cytokine release and reduce oxidative stress in several cell-based models. However, studies on the anti-inflammatory effects of black mustard seed extracts remain limited, particularly regarding cytokines involved in skin irritation and itch-associated inflammation, such as IL-6 and IL-31.

Therefore, the study aimed to investigate the biological activities, including antioxidant, antibacterial, and anti-inflammatory activities of black mustard seed extracts. Additionally, the anti-inflammatory activity of the extracts was evaluated in lipopolysaccharide (LPS)-stimulated RAW 264.7 macrophages by assessing the production of pro-inflammatory cytokines, including IL-6 and IL-31. Molecular docking was also performed to support the possible interactions between sinapic acid and inflammation-related targets. Overall, this study provides new insights into the potential application of black mustard seed extracts as a natural anti-inflammatory agent for skin-related and systemic inflammatory conditions, highlighting their added value as alternative therapeutic ingredients.

## 2. Results and Discussion

### 2.1. Extraction Yield of Black Mustard Seeds

**Figure 1:**
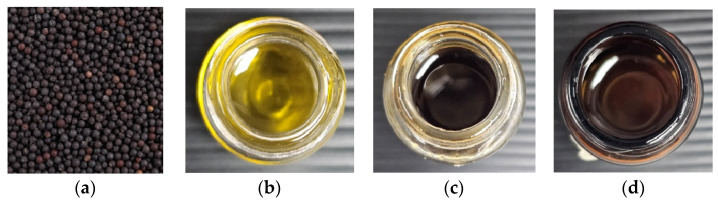
Physical appearance of (**a**) black mustard seeds, (**b**) hexane extract (HE), (**c**) ethanolic extract obtained from Soxhlet extraction (EE), and (**d**) ethanolic extract obtained from ultrasonication-assisted extraction (UE).

The extraction of black mustard seeds using different solvents and methods resulted in significantly different yields. Physical appearance of black mustard seeds and extracts are shown in Figure 1. The ethanolic extract (EE) obtained as a dark brown semi-solid, yielded 26.35 ± 2.67%, followed by the hexane extract (HE), which appeared as a yellowish oil, at 24.05 ± 2.00%. The ultrasonication extract (UE), characterized as a brown semi-solid produced the lowest yield at 19.48 ± 0.70%. The ethanolic and hexane extracts showed significantly higher yields than the ultrasonication extract (*p* < 0.05). The higher yield of the ethanolic extract may be attributed to its ability to dissolve a broad range of polar and semi-polar phytochemicals, including phenolics, flavonoids, and glucosinolates [12]. In contrast, hexane primarily extracts non-polar constituents such as essential oils and fatty acids. The lower yield observed for the ultrasonic-assisted ethanolic extraction be due to the rapid energy input and shorter extraction time, which could limit the complete release of phytochemicals from the seed matrix [13].

### 2.2. Analysis of Sinapic Acid Content in Black Mustard Seed Extracts

**Figure 2:**
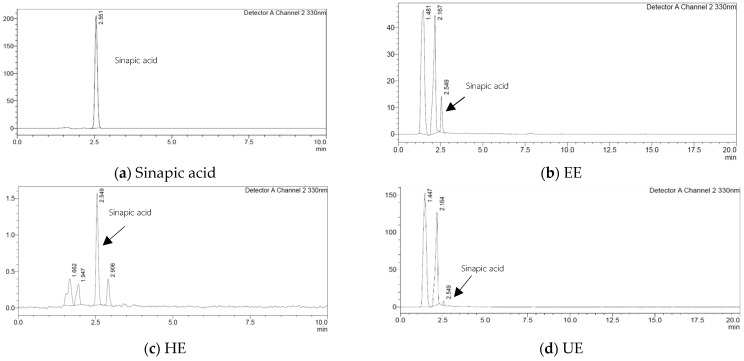
HPLC chromatograms of (**a**) sinapic acid, (**b**) black mustard seed ethanol extract (EE), (**c**) black mustard seed hexane extract (HE), and (**d**) black mustard seed ultrasonication ethanol extract (UE) detected using UV detection at a wavelength of 330 nm.

HPLC analysis confirmed that sinapic acid was found in all extracts but at markedly different concentrations, as shown in Figure 2. The sinapic acid standard exhibited a sharp peak at a retention time of 2.551 min. Quantification was performed using an injection concentration of 30 mg/L for each sample. According to the HPLC results, the EE showed the highest content of sinapic acid at 0.37 ± 0.15 mg sinapic acid/g extract and retention time at 2.549. The UE contained 0.13 ± 0.12 mg sinapic acid/g extract and retention time at 2.549, while the HE contained the lowest amount of sinapic acid at 0.06 ± 0.07 mg sinapic acid/g extract and retention time at 2.549. Black mustard seed are well known as a rich source of hydroxycinnamic acids, particularly sinapic acid and its derivatives, which represent phenolic acid group of the *Brassica* genus [14]. Previous studies have shown that sinapic acid and its esterified forms are the predominant phenolic acids in mustard seeds and related species, contributing significantly to their antioxidant, antibacterial and anti-inflammatory activities [15,16,17]. Sinapic acid has been frequently identified as the major phenolic ester in *Brassica* seeds, with high concentrations typically observed in ethanol-based extracts [18].

### 2.3. Total Phenolic and Total Flavonoid Contents of Black Mustard Seed Extracts

**Figure 3:**
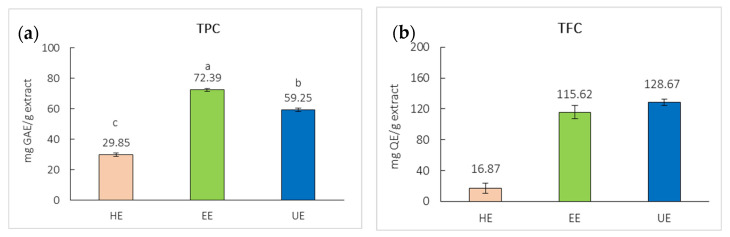
(**a**) TPC and (**b**) TFC of black mustard seed hexane extract (HE), black mustard seed ethanol extract (EE), black mustard seed ultrasonication ethanol extract (UE), and sinapic acid. Different letters (a–c) are significantly different at *p* < 0.05.

The total phenolic content (TPC) of the extracts is shown in Figure 3a. The EE exhibited the highest total phenolic content at 72.39 ± 0.09 mg gallic acid equivalent (GAE) per g extract, followed by the UE at 59.52 ± 1.68 mg GAE/g extract. The HE contained the lowest TPC (29.85 ± 0.63 mg GAE/g extract). The total flavonoid content (TFC) also differed significantly across extracts, as shown in Figure 3b. The UE showed the highest flavonoid content at 128.67 ± 3.90 mg quercetin equivalent (QE) per g extract, followed by EE at 115.62 ± 5.52 mg QE/g extract. The HE contained the lowest TFC (16.87 ± 7.53 mg QE/g extract). The markedly higher TPC observed in EE confirms the strong capacity of ethanol to solubilize phenolic compounds, particularly hydroxycinnamic acids such as sinapic acid and its derivatives, which are abundant in black mustard seeds.

### 2.4. Antioxidant Activity of Black Mustard Seed Extracts

**Figure 4:**
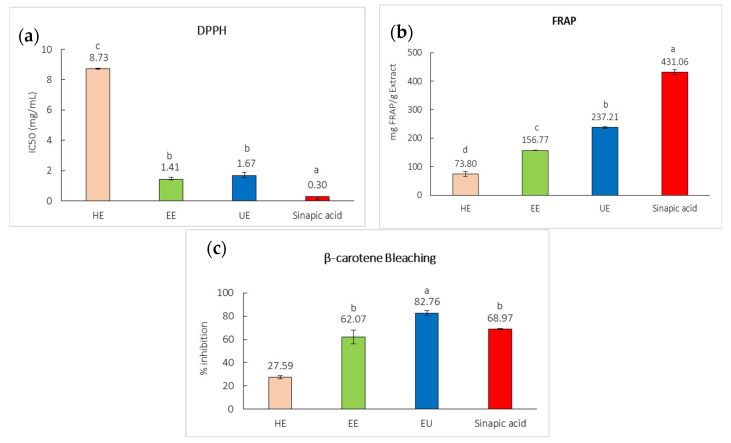
Antioxidant activity of black mustard seed hexane extract (HE), black mustard seed ethanol extract (EE), black mustard seed ultrasonication ethanol extract (UE), and sinapic acid when tested using (**a**) DPPH, (**b**) FRAP, and (**c**) β-carotene bleaching assay. Different letters (a–d) are significantly different at *p* < 0.05.

DPPH assay is a method that reflects the hydrogen-donating capacity of antioxidant compounds, while FRAP assay is used to evaluate the reducing power of bioactive compounds [19]. DPPH radical scavenging activity of the extracts and sinapic acid are shown in Figure 4a. EE and UE, with IC_50_ values of 1.41 ± 0.73 mg/mL and 1.67 ± 0.58 mg/mL, demonstrated significantly stronger free radical scavenging activity than HE (8.73 ± 0.08 mg/mL) (*p* < 0.05). This trend was consistent in the FRAP assay (Figure 4b), where UE exhibited the highest reducing power among the extracts (237.21 ± 6.15 mg FRAP/g extract), followed by EE (156.77 ± 6.64 mg FRAP/g extract), while HE displayed the lowest reducing power at 73.80 ± 4.78 mg FRAP/g extract (*p* < 0.05). Sinapic acid demonstrated the strongest reducing capacity overall. Previous studies reported strong antioxidant activity in ethanolic black mustard seed extracts, likely due to their higher phenolic and flavonoid contents, which further supports the role of sinapic acid as a key phenolic antioxidant with efficient hydrogen and electron donation capacity [20]. This may explain the strong DPPH radical scavenging and FRAP reducing activities observed in EE and UE.

The β-carotene bleaching assay reflects the ability of compounds to inhibit lipid peroxidation by preventing the oxidative degradation of β-carotene in a linoleic acid system [21]. The β-carotene bleaching results showed differences in antioxidant activity among the extracts and sinapic acid (Figure 4c). UE exhibited the highest lipid peroxidation inhibition at 82.76 ± 1.95% indicating that its activity was significantly higher than other extracts (*p* < 0.05). This was followed by EE (62.07 ± 5.97%) and sinapic acid (68.97 ± 0.34%), which showed significantly higher inhibition than HE (27.59 ± 1.34%). The strong antioxidant activity observed in EE and UE may be attributed to multiple mechanisms, including free radical scavenging, reducing power, and inhibition of lipid peroxidation. These antioxidant properties are closely associated with anti-inflammatory activity of the extracts, as oxidative stress play a critical role in the initiation and progression of inflammatory responses [8,22].

### 2.5. Antibacterial Activity of Black Mustard Seed Extracts

Based on the combined results of the bioactivity tests and HPLC analysis, EE and UE were chosen for further investigation, with sinapic acid identified as a potential key bioactive compound responsible for the observed effects of the extracts. Antibacterial activity using broth microdilution assay demonstrated that back mustard seed extracts exhibited inhibitory activity against skin-associated Gram-positive bacteria *S. epidermidis* as shown in Table 1. The EE and UE produced inhibition zones of 8 mm, while sinapic acid exhibited slightly stronger activity with an inhibition zone of 10 mm. In contrast, none of the extracts exhibited antibacterial activity against *S. aureus.* The extracts were further evaluated for minimal inhibitory concentration (MIC) and minimum bactericidal concentration (MBC) against *S. epidermidis* using broth microdilution assay. The results are shown in Table 2. Both EE and UE exhibited MIC values of 25 and 50 mg/mL, respectively, and both extracts showed identical MBC values of 50 mg/mL, confirming their bactericidal potential at higher concentrations. Sinapic acid demonstrated both MIC and MBC values of 6.25 mg/mL, indicating potent antibacterial activity against *S. epidermidis*.

In this study, the ability of EE and UE to inhibit *S. epidermidis* help to reduce bacteria-induced skin irritation and inflammation. Overgrowth of *S. epidermidis* on the skin can disturb skin microbiome and stimulate keratinocytes and immune cells to release inflammatory mediators, leading to redness, irritation, and pruritic response [23]. Previous study reported that black mustard seed extract from different extraction methods showed no inhibitory effect against *S. epidermidis* when evaluated using an agar disc diffusion method [24]. This difference may be attributed to variations in extraction techniques, resulting phytochemical compositions, and the higher extract concentrations used in the present study. Therefore, the antibacterial activity observed in EE and UE suggests that these extracts may be beneficial in reducing bacteria-related skin irritation and itch-associated inflammation. This effect may further help reduce inflammatory mediators involved in pruritic conditions, such as IL-6 and IL-31.

### 2.6. Lipoxygenase and Protease Inhibitory Effects of Black Mustard Seed Extracts

Lipoxygenases (LOX) are enzymes that catalyze the oxidation of arachidonic, linoleic, and other polyunsaturated fatty acids into a variety of bioactive metabolites. These metabolites play key roles in regulating inflammatory and immune responses and can contribute to the generation of reactive oxygen species (ROS) in the body [25]. In addition, LOX and their downstream signaling pathways have been implicated in the pathogenesis of several inflammatory disorders, including asthma, ulcerative colitis, and psoriasis [26].

Proteases are enzymes that hydrolyze peptide bonds and are classified into serine, cysteine, aspartic, and metalloproteases (MMPs). Among these, serine proteases are characterized by the presence of a serine residue at the active site, which plays a key role in peptide bond cleavage. These enzymes are critically involved in inflammatory processes and tissue remodeling [27]. This study employed serine protease as the model enzyme for evaluating protease inhibitory activity. Inhibition of protease activity can help prevent excessive protein degradation and consequently reduce the initiation and progression of inflammatory processes.

**Figure 5:**
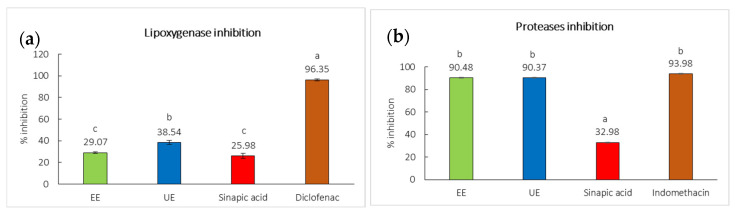
The anti-inflammatory activity of black mustard seed extracts against (**a**) lipoxygenases (LOX) compared to diclofenac sodium (positive control), and (**b**) proteases compared to indomethacin (positive control) and sinapic acid. All sample were tested at a concentration of 1 mg/mL. Different letters (a–c) are significantly different at *p* < 0.05.

This study evaluated the inhibitory effects of black mustard seed extracts in comparison with sinapic acid, diclofenac sodium, and indomethacin against LOX and protease enzymes. Given the established free radical scavenging capacity of the extracts, they may also function as effective inhibitors of LOX and protease activities. Among the extracts, UE demonstrated the highest LOX inhibitory activity at 38.54 ± 1.83, followed by EE (29.07 ± 0.66%) and sinapic acid (25.98 ± 2.25%), respectively. These values were markedly lower than that of the positive control, diclofenac sodium, which exhibited the strongest inhibition at 96.35 ± 0.94% (Figure 5a). Overall, UE displayed the greatest LOX inhibition among the extracts, although all extracts were considerably lower than diclofenac sodium. Interestingly, black mustard seed extracts exhibited comparable protease enzyme inhibition to the indomethacin (Figure 5b). Both EE and UE showed high and comparable protease inhibitory activity at 90.48 ± 0.10% and 90.37 ± 0.12%, respectively. In comparison, sinapic acid showed the lowest activity at 32.98 ± 0.21%. Previous study has reported that flavonoids can inhibit cyclooxygenase and lipoxygenase enzymes that reduce the formation of pro-inflammatory pain mediators [28].

### 2.7. Cytotoxicity of Black Mustard Seed Extracts on RAW 264.7 Cells

**Figure 6:**
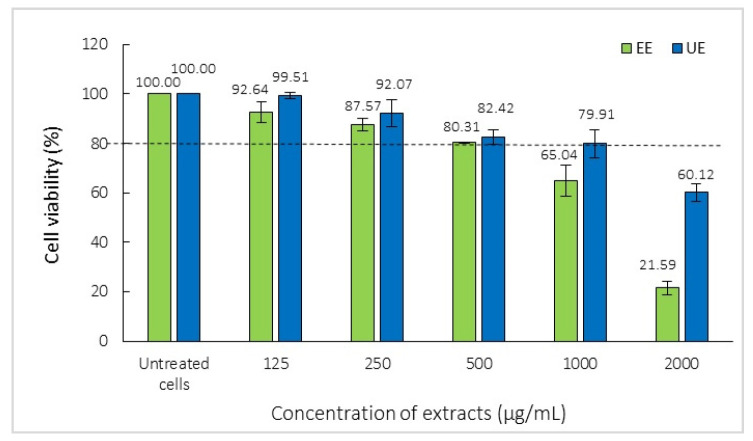
The cell viability of RAW 264.7 cells treated with black mustard seed extracts using MTT assays. The dotted line indicates the 80% cell viability threshold for non-cytotoxicity.

The effects of EE and UE on cell viability were evaluated using the MTT assay, as shown in Figure 6. Cells treated with vehicle only were defined as the control and set at 100% viability. The results showed that both EE and UE were non-cytotoxic at concentrations below 500 µg/mL, maintaining cell viability more than 80%. Which is a commonly accepted threshold for non-toxic conditions defined by ISO 10993-5 [29]. At higher concentrations, a dose-dependent decrease in cell viability was observed. For EE, cell viability decreased to 65.04% and 21.59% at 1000 and 2000 μg/mL, respectively, while UE showed higher viability at the same concentrations (79.91% and 60.12%, respectively) (*p* < 0.05). Based on these findings, non-cytotoxic concentrations of the EE and UE were chosen at 10 to 200 µg/mL for the anti-inflammatory activity study to minimize confounding effects of cytotoxicity.

### 2.8. Effect on Inhibition of Secretion and Gene Expression of Pro-Inflammatory Mediators

**Figure 7:**
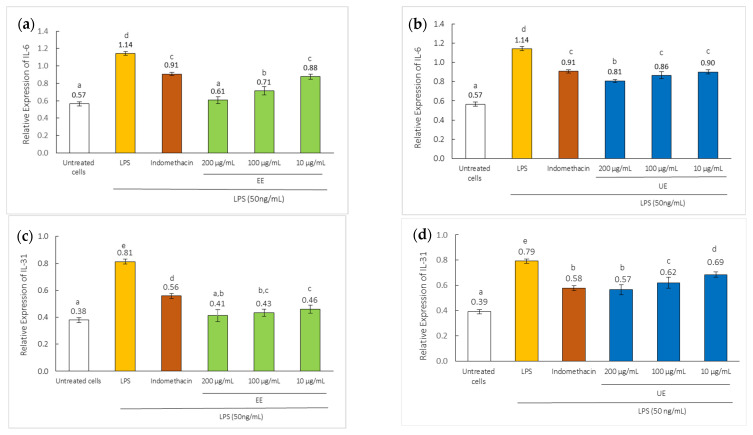
Effects of black mustard seed extracts on IL-6 [(**a**) EE, (**b**) UE] and IL-31 [(**c**) EE, (**d**) UE] mRNA expression in RAW 264.7 cells, compared to indomethacin (10 µg/mL). Different letters (a–e) are significantly different at *p* < 0.05.

LPS stimulation markedly increased the expression of IL-6 and IL-31 in RAW 264.7 cells, confirming the induction of an inflammatory response. Increased levels of IL-31, particularly in the skin, lungs, and stomach, are associated with allergic inflammation [9,30]. IL-31 is a recently identified cytokine belonging to the IL-6 family and plays a crucial role inflammatory process [30]. IL-31 is strongly implicated in atopic dermatitis, where its overexpression is strongly linked to pruritus and is considered a key mediator of itch [31]. In this study, LPS-stimulated RAW 264.7 macrophages exhibited significantly elevated levels of these cytokines, which were markedly suppressed following treatment with black mustard seed extracts, as shown in Figure 7. All extracts effectively reduced IL-6 and IL-31 expression in LPS-stimulated RAW 264.7 cells, with the strongest suppression observed at 200 µg/mL, indicating a concentration-dependent anti-inflammatory effect. At the same concentration of 10 µg/mL, both EE and UE showed IL-6 inhibitory effects comparable to those of indomethacin (10 µg/mL), the positive control, with no significant difference (*p* > 0.05). In addition, for IL-31, EE at 10 µg/mL exhibited significantly stronger suppression than indomethacin, whereas UE showed a weaker inhibitory effect.

The present findings demonstrated that EE and UE exerted anti-inflammatory effects by downregulating IL-6 and IL-31 expression. These results are consistent with previous studies reporting that mustard-derived phytochemicals reduce inflammatory cytokine production in macrophages through modulation of NF-κB/MAPK signaling pathways [32]. Sinapic acid, a major phenolic constituent in mustard seeds has been reported to suppress oxidative stress and inflammatory mediators, supporting its contribution to the cytokine inhibition observed in this study, particularly IL-31 [33]. Moreover, the antibacterial activity of EE and UE against *S. epidermidis* may further enhance their anti-inflammatory potential. Since excessive colonization of *S. epidermidis* can disrupt skin microbial balance and stimulate inflammatory mediator release, inhibition of this bacterium may help reduce microbe-associated inflammatory responses relevant to pruritic skin conditions [34].

**Figure 8:**
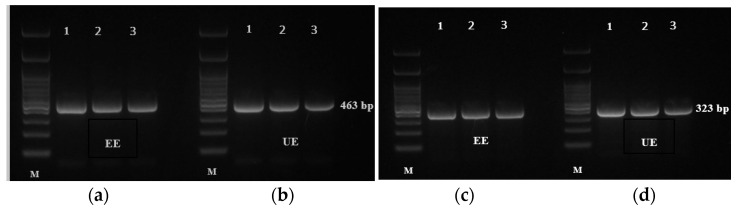
Agarose gel electrophoresis of IL-6 (463 bp) and IL-31 (323 bp) PCR products amplified from cDNA of LPS-stimulated RAW 264.7 cells treated with black mustard seed extracts. (**a**) IL-6 expression following treatment with EE, (**b**) IL-6 expression following treatment with UE, (**c**) IL-31 expression following treatment with EE, (**d**) IL-31 expression following treatment with UE. Lane 1: 10 µg/mL; Lane 2: 100 µg/mL; Lane 3: 200 µg/mL. M: 100-bp DNA ladder.

Consistent with the inhibitory effects of EE and UE on IL-6 and IL-31 expression observed in the qPCR analysis, these changes were further validated using agarose gel electrophoresis. RAW 264.7 cells were stimulated with LPS and then exposed to different concentrations of the extracts prior to amplification of IL-6 (463 bp) and IL-31 (323 bp) from cDNA. As shown in Figure 8, EE showed the strongest inhibition, with markedly lighter band intensities at 200 µg/mL, consistent with the qPCR results (IL-6: 0.61 ± 0.04; IL-31: 0.41 ± 0.04). UE also reduced IL-6 and IL-31 expression, though to a lesser extent, consistent with its moderate qPCR effects (IL-6: 0.81 ± 0.01; IL-31: 0.56 ± 0.04). These results indicate that EE exhibits significant anti-inflammatory activity. The reduction in mRNA expression levels suggests that the extracts exert their anti-inflammatory effects at the transcriptional level.

These results indicate that EE exhibited significant anti-inflammatory activity by downregulating IL-6 and IL-31 expression confirmed by both qPCR and agarose gel electrophoresis. However, the selection of IL-6 and IL-31 as target inflammatory markers was based on both macrophage-driven inflammation and itch-related skin cytokine responses. While IL-6 represents a classical NF-κB-dependent inflammatory marker, IL-31 is highly relevant to pruritus and inflammatory skin disorders, making these cytokines suitable biomarkers for assessing the inflammatory and itchy skin potential of the extracts [35,36].

### 2.9. Molecular Docking Analysis of Sinapic Acid Against Lipooxygenase-1 and Proteases

**Figure 9:**
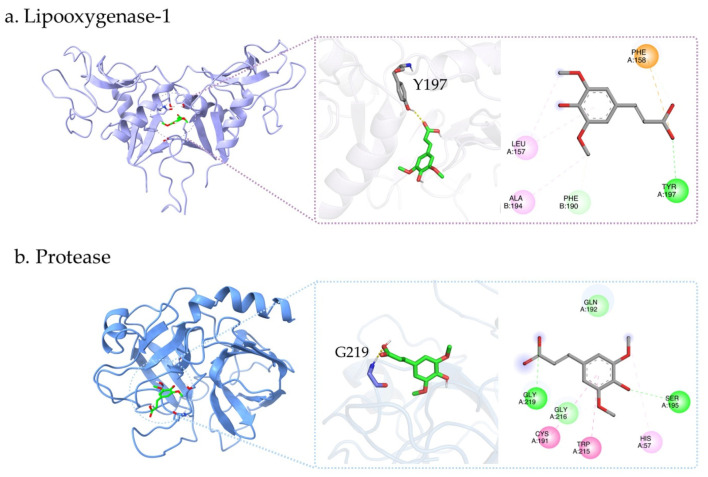
Molecular docking analysis of sinapic acid (green) in the active sites of (**a**) lipoxygenase-1 (purple ribbon) and (**b**) protease (blue ribbon). Green dashed lines indicate hydrogen bonds, pink represents hydrophobic interactions, and orange indicates π–anion interactions.

Molecular docking demonstrated that sinapic acid interacted with both lipoxygenase-1 and protease, with binding energies of −5.9 and −5.3 kcal/mol, respectively, indicating a more favorable binding affinity toward LOX-1. In the LOX-1 complex (Figure 9a), sinapic acid was positioned within the binding cavity, where its hydroxyl and carboxyl functional groups contributed to hydrogen bond formation with Tyr197. In addition, the aromatic scaffold of sinapic acid was surrounded by Leu157, Phe158, and Ala194, which likely enhanced ligand stabilization through hydrophobic contacts. These combined polar and hydrophobic interactions suggest that sinapic acid can be effectively accommodated within the LOX-1 binding pocket.

In the protease complex (Figure 9b), sinapic acid was located within the active-site region and formed hydrogen bond interactions with Gly219 and Ser195, while additional contacts with Gln192, Gly216, Cys191, Trp215, and His57 further stabilized the ligand within the pocket. The involvement of Ser195 and His57, which are associated with the catalytic environment of serine proteases [37], suggests that sinapic acid may interfere with substrate binding or catalytic activity through occupation of the active-site region. Overall, these findings indicate that sinapic acid may exert inhibitory potential against both targets through cooperative hydrogen bonding and hydrophobic interactions, with a relatively stronger affinity toward LOX-1.

## 3. Materials and Methods

### 3.1. Microorganisms

*Staphylococcus aureus* (*S. aureus*) (DMST 8840) and *Staphylococcus epidermidis* (*S. epidermidis*) (DMST 5868) were purchased from Department of Medical Sciences, Ministry of Public Health (Bangkok, Thailand).

### 3.2. Cell Line and Cytokines

The RAW 264.7 macrophage cell (TIB-71) was purchased from ATCC (Manassas, VA, USA). The PCR primers for β-actin, IL-6, and IL-31 were purchased from Eurofins MWG Operon, Konstanz, Germany.

### 3.3. Chemicals

Sinapic acid, indomethacin, diclofenac sodium, soybean lipoxygenase-1 (652,799 U/mg), and trypsin, a serine protease, were purchased from Sigma-Aldrich (St. Louis, MO, USA). 2,2-Diphenyl-1-picrylhydrazyl (DPPH), ferrous chloride (FeCl_2_), ferric chloride (FeCl_3_), ferrous sulfate (FeSO_4_), bovine serum albumin (BSA), Tris–hydrochloride (Tris-HCl), 3-(4,5-dimethylthiazol-2-yl)-2,5-diphenyl tetrazolium bromide (MTT), Folin–Ciocalteu reagent, aluminum chloride (AlCl_3_), lipopolysaccharide (LPS), linoleic acid, and β-carotene were also obtained from Sigma-Aldrich (St. Louis, MO, USA). Xylenol orange was purchased from QReC (Auckland, New Zealand). 2,4,6-Tris(2-pyridyl)-s-triazine (TPTZ) was purchased from Fluka (Buchs, Switzerland). Dulbecco’s Modified Eagle Medium (DMEM), fetal bovine serum (FBS), and penicillin–streptomycin were purchased from Gibco (Thermo Fisher Scientific, Waltham, MA, USA). Ethanol, dimethyl sulfoxide (DMSO), and hexane were purchased from Labscan Asia (Bangkok, Thailand). Other reagents, including TRIzol reagent and SYBR Green qPCR Master Mix were purchased from Gibthai (Bangkok, Thailand).

### 3.4. Roasting of Black Mustard Seeds Using an Air Fryer

Black mustard seed was purchased from Bangkok Crude Drugs Co., Ltd. (Bangkok, Thailand). Black mustard seeds were roasted according to the method described by Fadairo [38]. Briefly, black mustard seeds were preheated in an air fryer (MV-1350; SMARTHOME, Bangkok, Thailand) and roasted at 150 °C for 20 min. The roasted seeds were allowed to cool to room temperature and subsequently ground into fine powder using a blender (HR2221, 700W, Philips, Amsterdam, The Netherlands).

### 3.5. Black Mustard Seeds Extraction

#### 3.5.1. Soxhlet Extraction

Black mustard seed powder was extracted following the method of Stamenković [39]. The powder was extracted using a Soxhlet apparatus (BOECO, Hamburg, Germany) with hexane. The powder was subsequently dried in a hot air oven (Universal oven Memmert UN 55, Schwabach, Germany) at 60 °C for 1 h. Then, it was continuously extracted using a Soxhlet extraction apparatus with 95% (*v*/*v*) ethanol. After the extraction, the mixture was filtered through Whatman^®^ No. 1 filter paper (Cytiva, Marlborough, MA, USA) and the solvent was removed under vacuum using a rotary evaporator (Büchi R-200, Essen, Germany) until the constant weight was achieved. The obtained extracts were designated as black mustard seed hexane extract (HE) and back mustard seed ethanol extract (EE), respectively.

#### 3.5.2. Ultrasonication-Assisted Extraction

Black mustard seed powder was extracted using an ultrasonication-assisted extraction [13]. Briefly, 20 g of ground mustard seed powder was mixed with 100 mL 95% (*v*/*v*) ethanol. The mixture was subjected to ultrasonication for 30 min at a frequency of 40 kHz, with an ultrasound input power of 120 W and a heating power of 800 W, equipped with a digital timer and temperature controller. After extraction, the mixture was filtered through Whatman^®^ No. 1 filter paper, and the solvent was evaporated using a rotary evaporator (Büchi R-200, Germany) to obtain the black mustard seed ethanol extract (UE). The percentage yield of the extract was calculated using the following equation:

% yield = (E/P) × 100(1)

where E is the weight of the obtained extract and P is the weight of the plant powder used for extraction.

### 3.6. Analysis of Chemical Marker in Black Mustard Seed Extracts Using High-Performance Liquid Chromatography (HPLC)

Sinapic acid was used as chemical marker in the extracts. The content of sinapic acid in the extracts was analyzed using an HPLC (Shimadzu Prominence, Tokyo, Japan). Each sample (50 mg/L) was dissolved in methanol and filtered through a 0.45 μM polyvinylidene difluoride membrane filter before injection. The analysis of sinapic acid was performed according to the method described by Khattab [40] with some modifications. Chromatographic separation was carried out using a C18 column (SEG Analytical Science^®^, 4.6 × 150 mm, ENDURO C18G 125A, Trajan Scientific and Medical (Ringwood, VIC, Australia)) as a stationary phase. Mobile phase consisted of (A) acetonitrile and (B) 0.01% (*v*/*v*) formic acid (pH 3.0) with isocratic elution at a ratio of 70:30 (A:B). The flow rate and injection volume were set at 1.0 mL/min and 10 μL, respectively. HPLC chromatogram was detected using a UV detector (Shimadzu Prominence, Tokyo, Japan) at 330 nm. The experiment was performed in triplicates.

### 3.7. Determination of Total Phenolic Contents

Total phenolic content (TPC) of each extract was determined using a Folin–Ciocalteu assay with some modifications [41]. Briefly, the sample was mixed with Folin–Ciocalteu reagent (1:9; Folin–Ciocalteu reagent: distilled water) and incubated at room temperature for 5 min. Then, 7.5% (*w*/*v*) sodium carbonate solution was added to the mixture and incubated for 30 min in the dark. The absorbance was measured at 765 nm using a microplate reader (SPECTROstar Nano, Aylesbury, UK). The analytical curve was plotted using gallic acid at different concentrations, where y is the absorbance value and x is the content of gallic acid (mg). The results are expressed as milligrams of gallic acid equivalent (GAE) per gram of extract. The TPC was calculated using the equation below:

Total phenolic content (mg GAE/g extract) = (c × V × D)/N(2)

where c is the concentration of gallic acid (mg), V is the sample volume (mL), D is the dilution factor, and N is the weight of the sample (g).

### 3.8. Determination of Total Flavonoid Content

The total flavonoid content (TFC) of each extract was determined by an aluminum chloride colorimetric assay with some modifications [41]. Briefly, samples were mixed with 10% (*w*/*v*) aluminum chloride solution and 1 M potassium acetate. Then, the mixtures were kept at room temperature in the dark for 30 min. The absorbance was measured at 420 nm using a microplate reader (SPECTROstar Nano, Aylesbury, UK). The analytical curve was plotted using quercetin at different concentrations, where y is the absorbance value and x is the content of quercetin (mg). The results are expressed as milligrams of quercetin equivalent (QE) per gram of extract. The TFC was calculated using the equation below:

Total flavonoid content (mg QE/g extract) = (c × V × D)/N(3)

where c is the concentration of quercetin (mg), V is the sample volume (mL), D is the dilution factor, and N is the weight of the sample (g).

### 3.9. Determination of Antioxidant Activity

#### 3.9.1. 2,2-Diphenyl-1-picrylhydrazy (DPPH) Radical Scavenging Assay

The DPPH assay was performed according to the method of Nitthikan [42]. Each extract and sinapic acid were prepared in the range of 3.13–50 mg/mL. Briefly, 20 μL of sample were mixed with 180 μL of DPPH solution and incubated for 30 min at room temperature in the dark. The absorbance was measured at 520 nm using a microplate reader (SPECTROstar Nano, Aylesbury, UK). Sinapic acid was used as a standard antioxidant. The half-maximal inhibitory concentration (IC_50_) was calculated from the plotted linear graph of sample concentrations versus the percentage of inhibition.

#### 3.9.2. Ferric Reducing Antioxidant Power (FRAP) Assay

FRAP assay was used to evaluate the reducing property of the extracts following the method of Nitthikan [42]. The FRAP reagent was prepared as a mixture of 3 M acetate buffer (pH 3.6), 10 mM 2,4,6-Tripyridyl-S-triazine (TPTZ) dissolved in 40 mM of 37% (*v*/*v*) hydrochloric in deionized water, and 20 mM ferric chloride solution in a ratio of 50:5:5. Briefly, 0.20 μL of sample was added to a 96-well plate and mixed with 180 μL of the FRAP reagent for 5 min at room temperature. The dark blue solution was measured at 595 nm using a microplate reader (SPECTROstar Nano, Aylesbury, UK). Sinapic acid was used as a test compound. An analytical curve was plotted using ferrous sulfate at different concentrations as a standard curve. The results are presented as mg of ferrous sulfate equivalent per gram of extract or FRAP value that calculated from the following equation:

FRAP value (mg FeSO_4_/g of extract) = (c × V × D)/N (4)

where c is the concentration of ferrous sulfate (mg), V is the sample volume (mL), D is the dilution factor, and N is the weight of the sample (g).

#### 3.9.3. β-Carotene Bleaching Assay

β-carotene bleaching assay was used to evaluate the antioxidant activity of a substance by measuring its ability to prevent the oxidation of β-carotene following the method of Aree [43]. The β-carotene emulsion was prepared by dissolving β-carotene (5 mg), linoleic acid (20 mg), and Tween 80 (200 mg) in chloroform in a round bottom flask. The solvent was evaporated using a rotary evaporator at 40 °C for 10 min. The residue was then dispersed in 50 mL of deionized water and oxygenated for 30 min. Then, the mixture was vigorously shaken to form an emulsion. Next, 20 μL of sample was mixed with 180 μL of β-carotene emulsion in a 96-well plate. The absorbance was measured at 470 nm at 0, 30, 60, 90 and 120 min using a microplate reader (SPECTROstar Nano, Aylesbury, UK). Sinapic acid was used as a test compound. The percentage of inhibition was calculated using the following equation:

% Inhibition = (Acontrol − Asample)/Acontrol × 100(5)

where Acontrol is the absorbance of the reaction of deionized water and β-carotene emulsion and Asample is the absorbance of the reaction of sample solution and β-carotene emulsion.

### 3.10. Determination of Antibacterial Activity

The antibacterial activity of extracts against Gram-positive bacteria, *S. epidermidis* and *S. aureus* was evaluated using agar well diffusion assay, followed by determination of minimum inhibitory concentration (MIC) and minimum bactericidal concentration (MBC).

#### 3.10.1. Agar Well Diffusion Assay

The antibacterial activity of the extracts was evaluated using the agar disc diffusion method [44]. Briefly, bacterial strains were cultured on Mueller Hinton agar (MHA) for 24 h at 37 °C. The bacterial suspension was adjusted to 0.5 McFarland standard (1.5 × 10^8^ CFU/mL) and uniformly spread onto MHA plates using sterile cotton swabs. Wells of 6 mm diameter were aseptically punched into the agar using a sterile cork borer (Fisher Scientific, Loughborough, UK). Each well was filled with 50 µL of each extract and sinapic acid at a concentration of 100 mg/mL. Methanol (100%) was used as a negative control, while tetracycline (30 µg/mL) served as a positive control. The plates were incubated at 37 °C for 24 h. The diameter of the inhibition zone was measured in millimeters (mm). Antibacterial activity was expressed as the diameter of the clear zone.

#### 3.10.2. Determination of MIC and MBC

The MIC and MBC values of extracts were determined using the broth microdilution method according to the Clinical and Laboratory Standards Institute guideline (CLSI), 2024 [45]. Briefly, bacterial strains were cultured on MHA at 37 °C for 24 h. The bacterial suspension was adjusted to 0.5 McFarland standard (1.5 × 10^8^ CFU/mL). A volume of 100 µL of cation-adjusted Mueller-Hinton broth (CAMHB) was added to each well of a 96-well microplate. Each extract and sinapic acid were diluted in CAMHB to obtain concentrations of 100 mg/mL. Methanol and tetracycline (30 µg/mL) were used as negative and positive controls, respectively. Subsequently, 100 µL of bacterial suspension was added to each well. The plates were incubated at 37 °C for 18 h, and bacterial growth was assessed by measuring absorbance at 600 nm using a multimode microplate reader (TriStar^2^ LB 942, Berthold Technologies Bioanalytics, Bad Wildbad, Germany). Following MBC determination, 10 µL aliquots from wells showing no visible growth were plated onto fresh MHA plates and incubated at 37 °C for 24 h. The lowest concentration that resulted in no visible colony formation was recorded as the MBC.

### 3.11. Determination of Anti-Inflammatory Activity

#### 3.11.1. Lipoxygenase (LOX) Inhibitory Activity

The LOX inhibitory action was determined using the ferric oxidation of xylenol orange (FOX assay) according to some modified method [46]. The FOX reagent was prepared by mixing perchloric acid (110 mM), xylenol orange (150 μM), ferrous sulfate (2 mM) in methanol:water (9:1, *v*/*v*). Briefly, 50 µL of soybean LOX enzyme solution (100 ng protein/mL) in 50 mM Tris-HCl buffer (pH 7.4) was mixed with 20 μL of sample, and incubates at 25 °C for 5 min. The reaction was initiated by adding 50 μL of 140 μM linoleic acid in Tris-HCl buffer and incubated at 25 °C for 20 min in the dark. The reaction was terminated by adding 100 μL of FOX reagent. The absorbance of mixture was measured at 560 nm using a microplate reader (SPECTROstar Nano, Aylesbury, UK). Diclofenac sodium was used as a positive control. Sinapic acid was used as a test compound. LOX inhibition (%) was calculated using the following equation:

% Inhibition = (Acontrol − Asample)/Acontrol × 100(6)

where Acontrol is the absorbance of the reaction of enzyme, linoleic acid, and FOX reagent, and Asample is the absorbance of the reaction of sample, enzyme, linoleic acid, and FOX reagent.

#### 3.11.2. Proteases Inhibitory Activity

Proteases inhibition was determined based on the method of Preedalikit [46] with some modifications. The reaction mixture consisted of protease enzyme, Tris-HCl buffer (pH 7.4), and the sample. The mixture was incubated at 37 °C for 5 min in a water bath (Memmert Model W 600, Schwabach, Germany). Bovine serum albumin (BSA) at a concentration of 0.3% (*w*/*v*) was added as the substrate, and the mixture was further incubated at 37 °C for 20 min. The reaction was then terminated by adding perchloric acid 5% (*w*/*v*), resulting in protein precipitation. The absorbance was measured at 660 nm using a microplate reader (SPECTROstar Nano, Aylesbury, UK). Sinapic acid was used as a test compound. Indomethacin was used as a positive control. The level of proteases inhibition was calculated by comparing the sample to the untreated enzyme control. Proteases inhibition (%) was calculated as following equation:

Proteases inhibition (%) = [(Acontrol − Asample)/Acontrol] × 100(7)

where Acontrol is the absorbance of the reaction of the full reaction mixture without extract and Asample is the absorbance of the reaction of the full reaction mixture with extract.

### 3.12. Cell Culture

#### 3.12.1. Determination of Cytotoxicity

The cytotoxicity of the extracts on RAW 264.7 cells was evaluated using MTT assay [47]. In brief, RAW 264.7 cells were seeded at 1 × 10^4^ cells/well and incubated at 37 °C with 5% CO_2_ for 24 h. Then, the culture medium was then replaced with fresh medium containing sample solution, and the cells were further incubated under the same condition for 48 h. After incubation, 15 µL of the MTT reagent (5 mg/mL) was added and incubated for 4 h. The MTT solution was removed and 200 µL of DMSO was added to solubilize the purple formazan crystals. The solubilized formazan was measured at 570 nm with a reference wavelength of 630 nm using a microplate reader. The experiment was conducted in triplicate. The percentage of cell viability was calculated by the following equation:

% Cell viability = (ODsample well/ODvehicle control) × 100(8)

where the ODsample well is the absorbance of the treated cells. The ODvehicle control is the absorbance of the untreated cells.

#### 3.12.2. Inflammatory-Related Gene Expression Analysis

Inflammatory-related gene expression was evaluated using semi-quantitative reverse transcription polymerase chain reaction (RT-PCR). RAW 264.7 cells were overnight cultured in a 12-well plate. The 50 ng/mL of LPS was added, and the various concentrations of extract and positive control were treated and further incubated at 37 °C with 5% CO_2_ for 24 h. Total RNA was extracted from the treated cells using a RNA extraction kit (PureLink RNA Mini Kit, Invitrogen, Carlsbad, CA, USA). First-strand cDNA was synthesized using a reverse transcription kit (iScript cDNA Synthesis kit, Bio-Rad Laboratories, Hercules, CA, USA) and PCR was performed using Mytaq Red Mix kit (Bioline, London, UK) based on previously published method [48]. Amplification was carried out using gene-specific primers (Humanizing Genomics Macrogen, Seoul, Republic of Korea). The primer sequences were as follows: *β-actin*, 5′-TCATGAAGTGTGACGTTGACATCCGT-3′ (forward) and 5′-CCTAGAAGCATTTGCGGTGCACGATG-3′ (reverse); *IL-6*, 5′-CATCCAGTTGCCTTCTTGGGA-3′ (forward) and 5′-GCATTGGAAATTGGGGTAGGAAG-3′ (reverse); *IL-31*, 5′-GCCCGTCCAAGTCACTTCTT-3′ (forward) and 5′-GAAGATCAAAGTCCCGCCCA-3′ (reverse). PCR amplification was performed in a thermal cycler (Heal Force Classic K960-Thermo Cycler, Shanghai, China) under standard cycling conditions (30 cycles). The PCR products were separated by electrophoresis (Bio-rad, Hercules, CA, USA) on a 1.5% agarose gel (Invitrogen, Carlsbad, CA, USA), stained with a nucleic acid stain (Visafe Green, Gel Stain, Vivantis, Selangor, Malaysia), and visualized using a gel documentation system (Transilluminator Boi View, Bio Step, Jahnsdorf, Germany). Band intensities were quantified using gel image analysis software (ImageJ software version 1.54p), and the relative mRNA expression levels of inflammation-related genes were normalized to β-actin as an internal control.

### 3.13. Molecular Docking

Molecular docking was performed to investigate the binding interactions of sinapic acid with lipoxygenase-1 (LOX-1, PDB ID: 1YPQ) and serine protease (PDB ID: 1TRN). The three-dimensional structure of sinapic acid was obtained from the PubChem database and subjected to geometry optimization using Gaussian16 [49] at the B3LYP/6-31G(d,p) level. The crystal structures of LOX-1 and serine protease were retrieved from the RCSB Protein Data Bank. Protein preparation was carried out by removing water molecules and other non-essential heteroatoms, followed by the addition of polar hydrogen atoms. The prepared ligand and protein structures were converted into PDBQT format using AutoDockTools (version 1.5.7) [50]. Molecular docking simulations were conducted using AutoDock Vina version 1.2 [51], with the docking grid defined to cover the active-site region of each protein. To validate the docking protocol, the co-crystallized ligand was re-docked into the corresponding binding pocket, and a root-mean-square deviation (RMSD) value of less than 2.0 Å was considered acceptable. The binding pose with the lowest binding energy was selected as the most favorable conformation for further analysis. Protein–ligand interactions and binding modes were subsequently visualized using PyMOL (version 2.5) [52] and Discovery Studio Visualizer 2025.

### 3.14. Statistical Analysis

Data are presented as mean ± standard deviation (SD). All experiments were conducted in triplicate (*n* = 3). Statistical analysis was conducted using one-way analysis of variance (ANOVA), followed by Tukey’s post hoc test for multiple comparisons. All analyses were carried out using SPSS software (version 17.0), with statistical significance set at *p* < 0.05.

## 4. Conclusions

Black mustard seed extracts, particularly EE, exhibited significant antioxidant, antibacterial, and anti-inflammatory activities. Its ability to inhibit *S. epidermidis* supports its potential to reduce bacteria-associated inflammatory responses. Moreover, EE showed potential benefits in alleviating itch-associated inflammation through the downregulation of IL-6 and IL-31 expression. Molecular docking further supported sinapic acid as a key active compound, showing favorable binding interactions with inflammation-related targets, particularly lipoxygenase-1 and proteases, through key residues such as Tyr97 and Ser195. These findings suggest that sinapic acid may contribute to the observed enzyme inhibitory and anti-inflammatory activities. Therefore, EE provides new insights as a natural anti-inflammatory agent for skin-related and systemic inflammatory conditions, with added value as a potential alternative therapeutic option.

## Figures and Tables

**Table 1:** Antibacterial activity of black mustard seed ethanol extract (EE), black mustard seed ultrasonication ethanol extract (UE), and sinapic acid using agar well diffusion assay.

Concentration (mg/mL)	Inhibition Zone (mm.)				
	100			Methanol	Tetracycline (30 µg/mL)
Samples/ Microbial	EE	UE	Sinapic Acid		
*S. aureus*	6	6	6	6	44
*S. epidermidis*	8	8	10	6	44

**Table 2:** Minimum inhibitory concentration (MIC) and minimum bactericidal concentration (MBC) of black mustard seed ethanol extract (EE), black mustard seed ultrasonication ethanol extract (UE), and sinapic acid using broth microdilution assay.

Extracts/ Microbial	*S. epidermidis*	
	MIC (mg/mL)	MBC (mg/mL)
EE	25 ± 0	50
UE	50 ± 0	50
Sinapic acid	6.25 ± 0	6.25

## Data Availability

The original contributions presented in this study are included in the article. Further inquiries can be directed to the corresponding author.

## Abbreviations

The following abbreviations are used in this manuscript:

AITC	Allyl isothiocyanate
ANOVA	Analysis of variance
BSA	Bovine serum albumin
CAMHB	Cation-adjusted Mueller–Hinton broth
CFU	Colony-forming unit
COX-2	Cyclooxygenase-2
DMEM	Dulbecco’s Modified Eagle Medium
DMSO	Dimethyl sulfoxide
DPPH	2,2-Diphenyl-1-picrylhydrazyl
EE	Ethanolic extract
FBS	Fetal bovine serum
FRAP	Ferric reducing antioxidant power
GAE	Gallic acid equivalent
HE	Hexane extract
HPLC	High-performance liquid chromatography
IL-6	Interleukin-6
IL-31	Interleukin-31
IC_50_	Half-maximal inhibitory concentration
LPS	Lipopolysaccharide
LOX	Lipoxygenase
MBC	Minimum bactericidal concentration
MIC	Minimum inhibitory concentration
MMPs	Matrix metalloproteinases
MTT	3-(4,5-Dimethylthiazol-2-yl)-2,5-diphenyltetrazolium bromide
NF-κB	Nuclear factor kappa B
NO	Nitric oxide
PCR	Polymerase chain reaction
QE	Quercetin equivalent
RAW 264.7	Murine macrophage cell line
ROS	Reactive oxygen species
RT-PCR	Reverse transcription polymerase chain reaction
SD	Standard deviation
SPSS	Statistical package for the social sciences
TFC	Total flavonoid content
TNF-α	Tumor necrosis factor alpha
TPC	Total phenolic content
UE	Ultrasonication-assisted ethanolic extract

## References

[1] Alqaraawi S.S., Almujaydil M.S. (2025). Nutritional properties and potential therapeutic uses of *Brassica nigra*. Discov. Food.

[2] Rahman M., Khatun A., Liu L., Barkla B.J. (2018). Brassicaceae Mustards: Traditional and Agronomic Uses in Australia and New Zealand. Molecules.

[3] Duke J. (2012). Handbook of Legumes of World Economic Importance.

[4] Lietzow J. (2021). Biologically Active Compounds in Mustard Seeds: A Toxicological Perspective. Foods.

[5] Chen C. (2016). Sinapic Acid and Its Derivatives as Medicine in Oxidative Stress-Induced Diseases and Aging. Oxid. Med. Cell. Longev..

[6] Asaad N.K., Razooqi Q.A., Mustafa M.A. (2021). Toxicity of Cadmium Chloride on White Rats Liver and the Protective Role of *Brassica nigra* Seed Extract. Indian J. Forensic Med. Toxicol..

[7] Maqbool M., Chaudhary K., Chalotra R., Chauhan S., Dahiya R.S. (2024). Phyto-pharmacology of Most Common Indian Culinary Spices and their Potential in Developing New Pharmaceutical Therapies. Curr. Tradit. Med..

[8] Mittal M., Siddiqui M.R., Tran K., Reddy S.P., Malik A.B. (2014). Reactive oxygen species in inflammation and tissue injury. Antioxid. Redox Signal..

[9] Akira S., Uematsu S., Takeuchi O. (2006). Pathogen recognition and innate immunity. Cell.

[10] Williams M.R., Bagood M.D., Enroth T.J., Bunch Z.L., Jiang N., Liu E., Almoughrabie S., Khalil S., Li F., Brinton S. (2023). *Staphylococcus epidermidis* activates keratinocyte cytokine expression and promotes skin inflammation through the production of phenol-soluble modulins. Cell Rep..

[11] Rahman M., Khatun A., Liu L., Barkla B.J. (2024). Brassicaceae Mustards: Phytochemical Constituents, Pharmacological Effects, and Mechanisms of Action against Human Disease. Int. J. Mol. Sci..

[12] El Mannoubi I. (2023). Impact of different solvents on extraction yield, phenolic composition, in vitro antioxidant and antibacterial activities of deseeded *Opuntia stricta* fruit. J. Umm Al-Qura Univ. Appl. Sci..

[13] Rao M.V., Sengar A.S., K S.C., Rawson A. (2021). Ultrasonication-A green technology extraction technique for spices: A review. Trends Food Sci. Technol..

[14] Kozlowska H., Rotkiewicz D.A., Zadernowski R., Sosulski F.W. (1983). Phenolic acids in rapeseed and mustard. J. Am. Oil Chem. Soc..

[15] Chadni M., Flourat A.L., Reungoat V., Mouterde L.M.M., Allais F., Ioannou I. (2021). Selective Extraction of Sinapic Acid Derivatives from Mustard Seed Meal by Acting on pH: Toward a High Antioxidant Activity Rich Extract. Molecules.

[16] Meneguetti B.T., Machado L.D.S., Oshiro K.G.N., Nogueira M.L., Carvalho C.M.E., Franco O.L. (2017). Antimicrobial peptides from fruits and their potential use as biotechnological tools—A review and outlook. Front. Microbiol..

[17] Engels C., Schieber A., Gänzle M.G. (2012). Sinapic Acid Derivatives in Defatted Oriental Mustard (*Brassica juncea* L.) Seed Meal Extracts Using UHPLC-DAD-ESI-MS N and Identification of Compounds with Antibacterial Activity. Eur. Food Res. Technol..

[18] Gurgul A.A., Najjar Y., Chee A., An H., Che C.T., Park T.J., Warpeha K.M. (2023). Phenylpropanoid-enriched broccoli seedling extract can reduce inflammatory markers and pain behavior. J. Transl. Med..

[19] Gulcin I. (2025). Antioxidants: A comprehensive review. Arch. Toxicol..

[20] Rasera G.B., Hilkner M.H., de Alencar S.M., de Castro R.J.S. (2019). Biologically active compounds from white and black mustard grains: An optimization study for recovery and identification of phenolic antioxidants. Ind. Crops Prod..

[21] Nickavar B., Esbati N. (2012). Evaluation of the antioxidant capacity and phenolic content of three Thymus species. J. Acupunct. Meridian Stud..

[22] Reuter S., Gupta S.C., Chaturvedi M.M., Aggarwal B.B. (2010). Oxidative stress, inflammation, and cancer: How are they linked?. Free Radic. Biol. Med..

[23] Severn M.M., Horswill A.R. (2023). *Staphylococcus epidermidis* and its dual lifestyle in skin health and infection. Nat. Rev. Microbiol..

[24] Tunç K., Semerci A.B., Çınar E. (2020). Antibacterial and Antioxidant Activity of Some Seeds Used as Food. Food Health.

[25] Ribeiro D., Freitas M., Tomé S.M., Silva A.M.S., Porto G., Cabrita E.J., Marques M.M.B., Fernandes E. (2014). Inhibition of LOX by flavonoids: A structure-activity relationship study. Eur. J. Med. Chem..

[26] Mashima R., Okuyama T. (2015). The role of lipoxygenases in pathophysiology; new insights and future perspectives. Redox Biol..

[27] Eatemadi A., Aiyelabegan H.T., Negahdari B., Mazlomi M.A., Daraee H., Daraee N., Eatemadi R., Sadroddiny E. (2017). Role of protease and protease inhibitors in cancer pathogenesis and treatment. Biomed. Pharmacother..

[28] Khatun A., Rahman M., Uddin K.N., Ahsan K., Shimu S.N., Kobra K., Shimu S.A., Haque W., Rahman T., Jessy T.H. (2016). Preliminary study on thrombolytic property of thirty six different extracts of eight Bangladeshi medicinal plants with folkloric relevance. Orient. Pharm. Exp. Med..

[29] (2009). Biological Evaluation of Medical Devices—Part 5: Tests for In Vitro Cytotoxicity.

[30] Dillon S.R., Sprecher C., Hammond A., Bilsborough J., Rosenfeld-Franklin M., Presnell S.R., Haugen H.S., Maurer M., Harder B., Johnston J. (2004). Interleukin 31, a cytokine produced by activated T cells, induces dermatitis in mice. Nat. Immunol..

[31] Takamori A., Nambu A., Sato K., Yamaguchi S., Matsuda K., Numata T., Sugawara T., Yoshizaki T., Arae K., Morita H. (2018). IL-31 is crucial for induction of pruritus, but not inflammation, in contact hypersensitivity. Sci. Rep..

[32] Lee H.W., Lee C.G., Rhee D.K., Um S.H., Pyo S. (2017). Sinigrin inhibits production of inflammatory mediators by suppressing NF-κB/MAPK pathways or NLRP3 inflammasome activation in macrophages. Int. Immunopharmacol..

[33] Huang T., Zhao D., Lee S., Keum G., Yang H.O. (2023). Sinapic Acid Attenuates the Neuroinflammatory Response by Targeting AKT and MAPK in LPS-Activated Microglial Models. Biomol. Ther..

[34] D’Arcangelo S., Di Fermo P., Diban F., Ferrone V., D’Ercole S., Di Giulio M., Di Lodovico S. (2024). *Staphylococcus aureus*/*Staphylococcus epidermidis* from skin microbiota are balanced by Pomegranate peel extract: An eco-sustainable approach. PLoS ONE.

[35] Guttman-Yassky E., Irvine A.D., Brunner P.M., Kim B.S., Boguniewicz M., Parmentier J., Platt A.M., Kabashima K. (2023). The role of Janus kinase signaling in the pathology of atopic dermatitis. J. Allergy Clin. Immunol..

[36] Kim M., An J., Shin S.A., Moon S.Y., Kim M., Choi S., Kim H., Phi K.-H., Lee J.H., Youn U.J. (2024). Anti-inflammatory effects of TP1 in LPS-induced Raw264.7 macrophages. Appl. Biol. Chem..

[37] Ekici Ö.D., Paetzel M., Dalbey R.E. (2008). Unconventional serine proteases: Variations on the catalytic Ser/His/Asp triad configuration. Protein Sci..

[38] Fadairo O., Nandasiri R., Alashi A.M., Eskin N.A.M., Thiyam-Höllander U. (2021). Air frying pretreatment and the recovery of lipophilic sinapates from the oil fraction of mustard samples. J. Food Sci..

[39] Stamenković O.S., Djalović I.G., Kostić M.D., Mitrović P.M., Veljković V.B. (2018). Optimization and kinetic modeling of oil extraction from white mustard (*Sinapis alba* L.) seeds. Ind. Crops Prod..

[40] Khattab R., Eskin M., Aliani M., Thiyam U. (2010). Determination of Sinapic Acid Derivatives in Canola Extracts Using High-Performance Liquid Chromatography. J. Am. Oil Chem. Soc..

[41] Sembiring E.N., Elya B., Sauriasari R. (2018). Phytochemical screening, total flavonoid and total phenolic content and antioxidant activity of different parts of *Caesalpinia bonduc* (L.) Roxb. Pharmacogn. J..

[42] Nitthikan N., Leelapornpisid P., Natakankitkul S., Chaiyana W., Mueller M., Viernstein H., Kiattisin K. (2018). Improvement of stability and transdermal delivery of bioactive compounds in green robusta coffee beans extract loaded nanostructured lipid carriers. J. Nanotechnol..

[43] Aree T., Chaichit S., Junlatat J., Kiattisin K., Intharuksa A. (2025). Bridging Phytochemistry and Cosmetic Science: Molecular Insights into the Cosmeceutical Promise of *Crotalaria juncea* L.. Int. J. Mol. Sci..

[44] Nitthikan N., Preedalikit W., Supadej K., Chaichit S., Leelapornpisid P., Kiattisin K. (2024). Exploring the Wound Healing Potential of a *Cuscuta chinensis* Extract-Loaded Nanoemulsion-Based Gel. Pharmaceutics.

[45] Clinical and Laboratory Standards Institute (CLSI) (2024). Antimicrobial Disk Susceptibility Tests.

[46] Preedalikit W., Chittasupho C., Leelapornpisid P., Duangnin N., Kiattisin K. (2024). Potential of Coffee Cherry Pulp Extract against Polycyclic Aromatic Hydrocarbons in Air Pollution Induced Inflammation and Oxidative Stress for Topical Applications. Int. J. Mol. Sci..

[47] Nitthikan N., Leelapornpisid P., Naksuriya O., Intasai N., Kiattisin K. (2022). Potential and Alternative Bioactive Compounds from Brown *Agaricus bisporus* Mushroom Extracts for Xerosis Treatment. Sci. Pharm..

[48] Sripanidkulchai B., Junlatat J., Wara-aswapati N., Hormdee D. (2009). Anti-inflammatory effect of Streblus asper leaf extract in rats and its modulation on inflammation-associated genes expression in RAW 264.7 macrophage cells. J. Ethnopharmacol..

[49] Frisch M.J., Trucks G.W., Schlegel H.B., Scuseria G.E., Robb M.A., Cheeseman J.R., Scalmani G., Barone V., Petersson G.A., Nakatsuji H. (2016). Gaussian 16 Rev. B.01.

[50] Morris G.M., Huey R., Lindstrom W., Sanner M.F., Belew R.K., Goodsell D.S., Olson A.J. (2009). AutoDock4 and AutoDockTools4: Automated docking with selective receptor flexibility. J. Comput. Chem..

[51] Trott O., Olson A.J. (2010). AutoDock Vina: Improving the speed and accuracy of docking with a new scoring function, efficient optimization, and multithreading. J. Comput. Chem..

[52] Schrödinger, LLC (2015). PyMOL Molecular Graphics System.

